# Maternal and child nutrition services associated with nutritional knowledge and practices, India

**DOI:** 10.2471/BLT.22.289129

**Published:** 2023-10-31

**Authors:** Christopher T Andersen, Parvesh K Chopra, Niraj Dave, Deepali Hariprasad, Mohini Kak, Rahul Pandey, Devendra Tanwar, Deepika Nayar Chaudhery

**Affiliations:** a The World Bank, 1818 H Street NW, Washington, DC, 20433, United States of America.; b Nielsen India Private Limited; Gurgaon, India.; c The World Bank, New Delhi, India.; d The World Bank, Tunis, Tunisia.

## Abstract

**Objective:**

To evaluate whether maternal and child nutrition activities provided through the Indian Integrated Child Development Services scheme in India were associated with improved nutritional knowledge and practices among beneficiary women.

**Methods:**

We used a multistage sampling design to randomly select 4400 pregnant women or mothers of children younger than 2 years for a cross-sectional telephone survey. The respondents were beneficiaries of the scheme from across 11 Indian states. We used multivariate regression models controlling for sociodemographic factors to estimate the association between: scheme activities and nutrition messages heard; and scheme activities and nutrition practices. We also estimated the proportion of the total association with nutrition practices which was mediated by nutrition messages.

**Results:**

Among 110 regression models testing unique pairs of seven activities and 18 nutrition messages, 103 showed a statistically significant positive relationship (median risk ratio, RR: 1.14). For activities and nine nutrition practices, 39 out of 54 tested pairs were significantly associated (median RR: 1.16). We observed statistically significant mediation through nutrition messages for 28 out of 42 tested pairs of activities and nutrition practices.

**Conclusion:**

Receipt of the scheme’s activities was associated with improved nutrition knowledge and practices. Improvements in practices were statistically mediated by improvements in knowledge. These findings suggest that a large-scale nutrition scheme with a strong counselling component could successfully change beneficiary behaviours.

## Résumé

**Objectif:**

Déterminer si les activités de nutrition de la mère et de l'enfant menées dans le cadre du programme Integrated Child Development Services en Inde entraînent une amélioration des connaissances et pratiques nutritionnelles chez les femmes qui en bénéficient.

**Méthodes:**

Nous avons employé un système d'échantillonnage à plusieurs degrés pour sélectionner au hasard 4400 femmes enceintes ou mères d'enfants de moins de deux ans en vue d'effectuer une enquête téléphonique transversale. Les participantes étaient réparties dans 11 États indiens et bénéficiaient toutes du programme. Nous avons appliqué des modèles de régression multivariée pour tenir compte des facteurs sociodémographiques et identifier le lien entre: les activités du programme et les messages concernant la nutrition d'une part; les activités du programme et les pratiques nutritionnelles de l'autre. Nous avons également estimé le pourcentage que représentait ce lien avec les pratiques nutritionnelles dans sa globalité, transmis par le biais des messages délivrés à ce sujet.

**Résultats:**

Parmi 110 modèles de régression ayant testé des paires uniques de sept activités et 18 messages relatifs à la nutrition, 103 ont montré un lien positif statistiquement significatif (risque relatif médian, RR: 1,14). Pour certaines activités et neuf pratiques nutritionnelles, 39 des 54 paires examinées étaient étroitement liées (RR médian: 1,16). Enfin, nous avons constaté une intervention statistiquement significative des messages de nutrition pour 28 des 42 paires d'activités et de pratiques nutritionnelles étudiées.

**Conclusion:**

La participation aux activités du programme a permis de développer de meilleures connaissances et pratiques nutritionnelles. L'amélioration des pratiques était statistiquement favorisée par l'amélioration des connaissances. Ces résultats suggèrent qu'un programme de nutrition à large échelle accompagné d'une série de conseils aux bénéficiaires pourrait réellement faire évoluer leur comportement.

## Resumen

**Objetivo:**

Evaluar si las actividades de nutrición maternoinfantil que se ofrecen a través del plan de Servicios Integrados de Desarrollo Infantil de la India se asocian a una mejora de los conocimientos y prácticas nutricionales de las mujeres beneficiarias.

**Métodos:**

Se utilizó un diseño de muestreo polietápico para seleccionar aleatoriamente a 4400 mujeres embarazadas o madres de niños menores de 2 años para una encuesta telefónica transversal. Las encuestadas eran beneficiarias del plan de 11 estados de la India. Se utilizaron modelos de regresión multivariante que controlaban los factores sociodemográficos para estimar la asociación entre: las actividades del plan y los mensajes sobre nutrición escuchados; y las actividades del plan y las prácticas de nutrición. También se calculó el porcentaje de la asociación total con las prácticas nutricionales que estaba mediada por los mensajes sobre nutrición.

**Resultados:**

Entre los 110 modelos de regresión que probaron pares únicos de siete actividades y 18 mensajes de nutrición, 103 mostraron una relación positiva estadísticamente significativa (mediana de la razón de riesgo, RR: 1,14). En el caso de las actividades y nueve prácticas nutricionales, 39 de los 54 pares probados mostraron una relación significativa (mediana de la RR: 1,16). Se observó una mediación estadísticamente significativa a través de los mensajes de nutrición en 28 de los 42 pares evaluados de actividades y prácticas de nutrición.

**Conclusión:**

La participación en las actividades del plan se asoció a la mejora de los conocimientos y las prácticas en materia de nutrición. Las mejoras en las prácticas estuvieron mediadas estadísticamente por las mejoras en los conocimientos. Estos resultados sugieren que un plan de nutrición a gran escala con un fuerte componente de asesoramiento podría cambiar con éxito los comportamientos de los beneficiarios.

## ملخص

الغرض تقييم أنشطة تغذية الأم والطفل المقدمة من خلال المخطط الهندي المتكامل لتنمية الطفل في الهند، وهل هي مرتبطة بتحسين المعرفة والممارسات الغذائية بين النساء المستفيدات.

الطريقة قمنا باستخدام تصميم لجمع العينات متعدد المراحل ليتم انتقاء 4400 امرأة حامل أو أم لأطفال تقل أعمارهم عن سنتين، بشكل عشوائي، لإجراء مسح مقطعي عبر الهاتف. وكان المشاركون من المستفيدين من المخطط من 11 ولاية هندية. استخدمنا نماذج التحوف متعددة المتغيرات التي تتحكم في العوامل الاجتماعية السكانية لتقدير الارتباط بين: أنشطة المخطط ورسائل التغذية المسموعة؛ وأنشطة المخطط والممارسات الغذائية. لقد قمنا أيضًا بتقدير نسبة الارتباط الإجمالي بممارسات التغذية التي توسطت فيها رسائل التغذية.

النتائج من بين 110 نموذجًا للتحوف، تختبر أزواجًا فريدة من سبعة أنشطة و18 رسالة غذائية، أظهر 103 منها علاقة إيجابية ذات دلالة إحصائية (متوسط نسبة المخاطر: 1.14). بالنسبة للأنشطة وتسع ممارسات غذائية، فإن 39 زوجًا من أصل 54 زوجًا تم اختبارها كانت مرتبطة بشكل كبير (متوسط نسبة المخاطر: 1.16). لاحظنا وجود توسط إحصائي ملموس خلال رسائل التغذية لعدد 28 من أصل 42 زوجًا من الأنشطة وممارسات التغذية التي تم اختبارها.

الاستنتاج ارتبط تلقي أنشطة المخطط بتحسين المعرفة والممارسات الغذائية. وكانت التحسينات في الممارسات بوساطة إحصائية من خلال التحسينات في المعرفة. تشير هذه النتائج إلى أن مخطط التغذية واسعة النطاق التي تحتوي على مكون استشاري قوي يمكنه أن يغيّر سلوكيات المستفيدين بنجاح.

## 摘要

**目的:**

评估通过“印度儿童发展综合性服务计划”在印度开展的母婴营养活动是否与受益妇女营养知识水平的提高和实践的改善有关。

**方法:**

我们采用多阶段抽样设计,随机抽取了 4,400 名孕妇或 2 岁以下儿童的母亲,对其进行横断面电话调查。受访者是来自印度 11 个邦的计划受益者。我们使用控制社会人口因素的多元回归模型来评估通过该计划开展的活动与受益者了解到的营养信息之间的关联以及通过该计划开展的活动与其营养实践之间的关联。我们还估计了由营养信息所介导的营养实践的总关联比例。

**结果:**

在 110 个测试 7 种活动和 18 种营养信息唯一配对的回归模型中,103 个模型显示出具有统计学意义上的显著正相关关系(风险比中位数,RR:1.14)。对于活动和 9 种营养实践,54 对测试中有 39 对显示显著相关关系(RR 中位数:1.16)。我们观察到,在 42 对被测试的活动和营养实践中,有 28 对在统计学意义上与营养信息有的显著介导关系。

**结论:**

通过该计划开展的活动与营养知识水平的提高和实践的改善有关。实践的改善在统计学意义上是由知识水平的提高所介导的。这些测试结果表明,提供专业咨询服务的大型营养活动计划可以有效改变受益人的行为。

## Резюме

**Цель:**

Оценить, насколько мероприятия в области питания матери и ребенка, проводимые в рамках индийской программы комплексного обслуживания детей в Индии, связаны с улучшением знаний и практики питания среди женщин, получающих пособие.

**Методы:**

Для проведения перекрестного телефонного опроса использовалась многоступенчатая схема случайного отбора 4400 беременных женщин или матерей детей в возрасте до 2 лет. Респондентами были получатели пособий программы из 11 штатов Индии. Для оценки связи между деятельностью в рамках программы и полученными сообщениями о питании, деятельностью в рамках программы и практикой питания использовались многомерные регрессионные модели, контролирующие социально-демографические факторы. Также оценивалась доля общей связи с практикой питания, которая была опосредована сообщениями о питании.

**Результаты:**

Из 110 регрессионных моделей при анализе уникальных пар из семи мероприятий и 18 сообщений о питании 103 показали статистически значимую положительную связь (медиана отношения рисков, ОР: 1,14). Для мероприятий и девяти практик в области питания 39 из 54 анализируемых пар были значимо связаны (медиана ОР: 1,16). В отношении 28 из 42 анализируемых пар мероприятий и практик в области питания наблюдалось статистически значимое опосредование через сообщения о питании.

**Вывод:**

Получение услуг в рамках программы было связано с улучшением знаний и практики в области питания. Улучшение практической деятельности было статистически опосредовано улучшением знаний. Эти результаты свидетельствуют о том, что крупномасштабная программа в области питания с активной консультационной составляющей может способствовать изменению поведения получателей пособий.

## Introduction

Undernutrition is a major challenge in India. In 2019–2021, an estimated 35.5% of children younger than 5 years were stunted, 19.3% were wasted and 67.1% were anaemic.^1^ Among women of reproductive age, an estimated 57.0% were anaemic.^1^ Evidence shows that appropriate nutrition practices reduce the prevalence of undernutrition.^2^ However, in India appropriate nutrition practices could be considerably improved. For example, in 2019–2021, an estimated 36.3% of children younger than 6 months were not exclusively breastfed, 54.1% of children aged 6–8 months were not receiving solid or semi-solid food and breast milk, and 88.7% of children aged 6–23 months were not receiving an adequate diet.^1^ Additionally, an estimated 55.9% of women did not consume iron-folic acid for 100 days or more during their last pregnancy.^1^

**Fig. 1:**
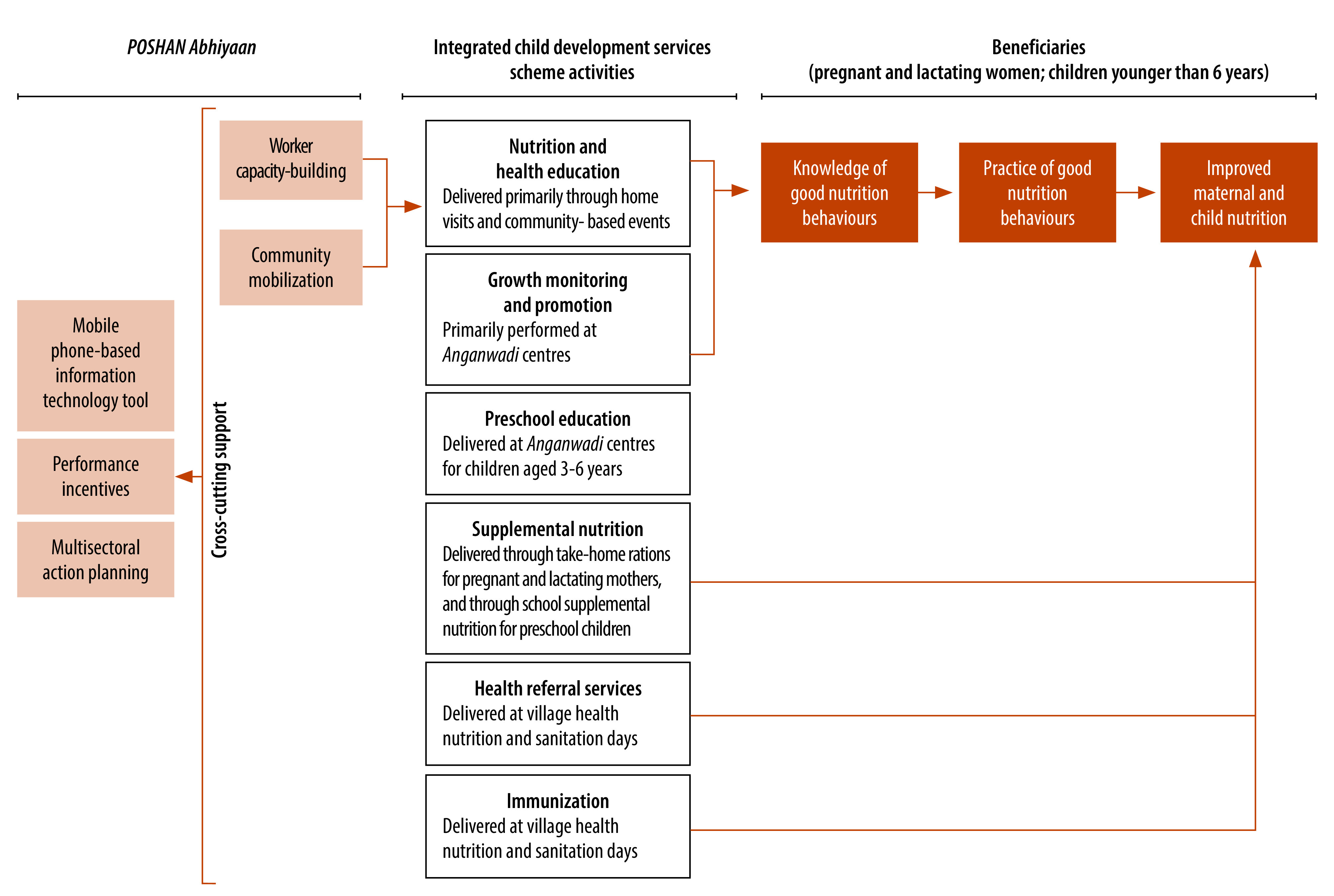
Theory of change linking activities of the Integrated Child Development Services scheme to child nutritional outcomes, India

The Integrated Child Development Services scheme (hereafter the scheme), delivered by the Indian Ministry of Women and Child Development, is a national nutrition programme that aims to reduce maternal and child undernutrition.^3^ The scheme delivers six core activities: nutrition and health education; supplementary nutrition; growth monitoring and promotion; pre-school non-formal education; immunization; and health referral services. In 2018, the Prime Minister’s Overarching Scheme for Holistic Nourishment (*POSHAN Abhiyaan*) introduced new initiatives to the scheme.^4^ The new initiatives were: use of a mobile phone-based information technology tool, called ICDS-CAS; capacity-building of the scheme’s frontline *Anganwadi* workers; community mobilization and nutrition behaviour change communication; performance-based incentives for *Anganwadi* workers; and multisectoral action planning. The theory of change postulates that for the scheme's activities to affect child health outcomes, they must first reach the beneficiaries. These activities can then either directly influence nutritional outcomes, like through nutrition supplementation; or indirectly by enhancing knowledge, which is then presumed to translate into improved practices (Fig. 1).

Approximately 1.4 million *Anganwadi* workers deliver a set of complementary activities to over 80 million beneficiaries, including both pregnant and lactating women as well as children aged 0–6 years.^5^ During home visits, *Anganwadi* workers provide nutrition counselling to pregnant and lactating women.^6^ These visits are scheduled according to a standardized home visit planner; *Anganwadi* workers should make a total of 13 home visits during pregnancy and the first 6 months postpartum. Furthermore, they deliver supplemental feedings, known as take-home rations, to pregnant and lactating women and children aged 7 months to 3 years, every two to four weeks. Take-home rations comprise a variety of products, dry rations and hot cooked meals, depending on the implementation locality.^7^ Beneficiaries should visit *Anganwadi* centres on a monthly basis for planned growth monitoring and promotion visits for children.^8^ During these visits, *Anganwadi* workers identify malnourished children and provide counselling to caregivers on appropriate feeding practices. The *Anganwadi* centres also provide pre-school education to children aged 3–6 years.^5^ Immunization and health referral activities primarily occur through monthly village health nutrition and sanitation days at the *Anganwadi* centre or at the subhealth centre. The local government health personnel support these days.^5^

Previous studies have assessed nutritional outcomes of participants of the scheme. Three studies suggest that nutritional outcomes have not improved among beneficiaries,^9^^–^^11^ while another study suggests that service utilization is linked to improved weight for age.^12^ One nationally representative study noted that beneficiaries had more diverse diets relative to a control group.^11^ From an implementation perspective, to discern why activities might not lead to improved nutrition, it is essential to understand the intermediary steps involving nutritional knowledge and practices. We therefore aimed to estimate the association of all major nutritional activities delivered through the scheme (under the enhanced *POSHAN Abhiyaan* approach) to pregnant and lactating mothers and their children with nutrition knowledge and practices among the beneficiaries. We also assessed statistical mediation of improved knowledge on practices. 

## Methods

Between 6 March and 22 April 2021, we conducted a cross-sectional telephone survey. The survey was telephonic due to the ongoing coronavirus disease 2019 (COVID-19) pandemic. We interviewed scheme beneficiaries, residing in one of the 11 states designated as priority states by the Indian government under *POSHAN Abhiyaan*, who were either pregnant or had children younger than two years. These states were Andhra Pradesh, Bihar, Chhattisgarh, Gujarat, Jharkhand, Karnataka, Madhya Pradesh, Maharashtra, Rajasthan, Tamil Nadu and Uttar Pradesh. These 11 states account for 82% of the scheme’s total beneficiaries. 

All beneficiaries were enrolled in activities at an *Anganwadi* centre. The centres adapted the activities to the COVID-19 pandemic context: centres were closed in most states, and *Anganwadi* workers were instead performing home visits twice per month to deliver counselling and take-home rations. Group education and service delivery activities varied by state; they were either discontinued or conducted with limited participation. Despite these changes, the recall period used for this survey (either one month or one year, depending on the service) encompasses a period where all activities were available.

We used a multistage cluster sampling design at the district and *Anganwadi* centre levels for participant selection (detailed methods are available in the online repository).^13^ Once *Anganwadi* centres were selected, beneficiaries were contacted using computer-assisted simple random selection until the pre-specified sample size of 4400 participants (400 per state) was reached.

### Data collection

Data collection tools included questions on beneficiary sociodemographic information, scheme activities received, nutrition messages that the beneficiary had heard and their nutrition practices. We designed questions on nutrition practices to match the language used in prior national surveys to ensure consistency.^14^^,^^15^ In alignment with World Health Organization (WHO) guidance, participants self-reported nutrition practices.^16^ Using a computer-assisted telephonic interview platform, female interviewers conducted the survey in six local languages: Gujarati, Hindi, Kannada, Marathi, Tamil, and Telugu. A description of training and quality assurance measures is available in the online repository.^13^

We collected sociodemographic variables and categorized them in alignment with India’s National Family and Health Survey.^1^ In the analysis, we used seven sociodemographic indicators: age at time of interview; age at time of marriage; highest year of education achieved; religion; caste or tribe; whether the household has a below poverty line card; and number of children. We did not attempt to assess the gender of the participants, nor the sex of the children. Further information is available in the online repository.^13^

We asked the respondents how many days’ worth of take-home rations they had received in the past month. A binary variable was calculated to indicate those respondents who had received 21 days or more worth of rations. Interviewers asked all women whether they had received nutrition information from an *Anganwadi* worker during their pregnancy, and pregnant women were asked if an *Anganwadi* worker had visited their home in the past month. Respondents were asked whether they had ever attended a community-based event, which is a group celebration of milestones in the mother or child’s life which includes nutrition messages. They were also queried if they had attended a village health sanitation and nutrition day in the past month. This monthly event at the *Anganwadi* centre focuses on delivery of basic activities such as vaccinations and health information. The mothers were also asked whether they had taken their child for growth monitoring (linked to nutrition messages on infant and young child feeding) in the past year. Finally, we used a binary variable to indicate respondents who lived in districts where the mobile application ICDS-CAS was under implementation.

We asked all respondents what nutrition messages they had previously heard, and they were prompted with a list of key messages that are included in the scheme’s nutrition counselling curriculum. Respondents were asked if they had ever received each nutrition message; we did not assess the frequency, timing and context in which they had received the message. We used binary variables to indicate respondents who had received the message. For the purposes of this analysis, nutrition messages received by participants are used to indicate nutrition knowledge.

We used binary variables of nutrition practices, as defined by WHO, the Food and Agriculture Organization or Indian national protocols.^16^^–^^19^ We calculated the following indicators: consumption of 100 or more days’ of iron-folic acid supplements during the last pregnancy; achievement of the minimum dietary diversity among pregnant women; breastfeeding in the first hour of life; exclusive breastfeeding among children younger than 6 months; consumption of solid, semi-solid or soft food and breast milk among children aged 6–8 months; minimum dietary diversity, minimum meal frequency and minimum acceptable diet among children aged 6–23 months; and whether the mother increased food or breastfeeding quantity after a child’s illness.

### Data analysis

We used Stata software, version 15 (StataCorp LLC., College Station, United States of America) to analyse data. To account for state population size (projected 2021 population among women aged 15–49 years),^20^ we weighted point estimates. We corrected confidence intervals (CIs) to account for the clustered sampling design, finite population sampling and stratification.

We calculated proportions for sociodemographic variables, scheme activities, nutrition messages received and nutrition practices. Using Poisson regressions with robust standard errors,^21^ we calculated risk ratios (RRs) for (i) the association between activities and nutrition messages received; and (ii) the association between activities and nutrition practices. To estimate the proportion of the total direct effect between activities provided and nutrition practices which are mediated by nutrition messages received, we conducted mediation analyses.^22^ To summarize the contribution of activities across multiple messages and practices, median RRs and median proportions mediated were calculated. A description of the regression models, mediation algorithm, and handling of missing data is available in the online repository.^13^ We considered *P*-values below 0.05 statistically significant.

### Ethical considerations

The Ministry of Women and Child Development, Government of India granted permission to conduct the study. We obtained verbal informed consent from each participant before the interview. Considerations related to confidentiality and participant risk are presented in the online repository.^13^

## Results

Out of 880 *Anganwadi* centres selected for sampling, beneficiary contact information was received for 745 (84.7%) centres. We obtained phone numbers for 20 244 beneficiaries, of which 16 145 were selected for contact using computer-assisted simple random selection. Of those selected, 8874 (55.0%) had phones that were unreachable, leaving 7271 available contacts. Among those successfully contacted, 995 were incorrect numbers, 1258 were not available for interview, 454 were no longer eligible for the survey and 164 refused to participate. The final sample size was 4400 respondents.

The sample population was relatively young, with 81.8% (after population weighting) of respondents being between 20 and 29 years old at the time of interview. The majority (53.0%) of respondents got married before the age of 20 years. Many participants were from disadvantaged backgrounds, including 53.2% living in households with a below-poverty line card; 31.0% from scheduled castes or tribes; and 19.6% of respondents having only primary school education (Table 1).

**Table 1:** Sociodemographic characteristics of study participants receiving *Anganwadi* activities in 11 states, India, 2021

Characteristic	Weighted % (95% CI) (*n* = 4400)^a^
**Age of participant, years**	
< 20	4.9 (4.1 to 5.9)
20–24	45.6 (42.9 to 48.2)
25–29	36.2 (33.9 to 38.5)
≥ 30	13.3 (11.9 to 14.8)
**Age at marriage, years**	
< 15	2.3 (1.7 to 3.2)
15–19	50.7 (47.4 to 54.1)
20–24	38.8 (36.2 to 41.5)
≥ 25	8.2 (6.7 to 9.9)
**Education**	
No education	11.3 (9.9 to 12.9)
Primary school (grades 1–5)	8.3 (7.3 to 9.6)
Middle school (grades 6–8)	14.8 (13.5 to 16.3)
Junior high school (grades 9–10)	22.4 (20.8 to 24.2)
Senior high school (grades 11–12)	20.9 (19.0 to 23.0)
More than high school	22.2 (20.0 to 24.5)
**Religion**	
Hindu	87.4 (83.7 to 90.3)
Muslim	9.6 (6.9 to 13.3)
Other	3.0 (2.2 to 4.0)
**Caste or tribe**	
Scheduled caste or tribe	31.0 (27.9 to 34.3)
Other backward class	46.0 (42.4 to 49.7)
General class	21.0 (18.0 to 24.4)
Do not know	1.9 (1.2 to 3.2)
**Household has below poverty line card**	
Yes	53.2 (49.6 to 56.7)
No	43.8 (40.4 to 47.3)
Do not know	3.0 (2.3 to 3.8)
**No. of children**	
None	10.7 (9.7 to 11.8)
One child	41.4 (39.6 to 43.2)
Two children	30.3 (28.4 to 32.3)
Three or more children	17.6 (15.5 to 19.8)

CI: confidence interval.

^a^ Proportion is survey weighted and CIs are adjusted for clustered sampling design.

The most frequently reported service received in the past year was growth monitoring and promotion activities (Table 2; weighted 73.7%). Sixty-two percent of respondents indicated that they had received nutrition information from the *Anganwadi* worker during pregnancy. About half of the respondents (55.4%) had received more than 21 days’ worth of take-home rations. Group events, such as community-based events and village health sanitation and nutrition days, were the activities least frequently reported.

**Table 2:** Activities of the Integrated Child Development Services scheme, nutrition messages received, and nutrition practices among study participants in 11 states, India, 2021

Activity	Weighted % (95% CI)^a^
**Scheme activity**	
Take-home ration received for 21 days or more in the past month^b^ (*n* = all 4400 participants)	55.4 (50.5 to 60.1)
Information on nutrition received from an *Anganwadi* worker during pregnancy (*n* = all 4400 participants)	62.3 (58.5 to 66.1)
*Anganwadi* worker visited the home in the past month (*n* = 1100 pregnant women)	65.5 (60.6 to 70.0)
Ever attended a community-based event (*n* = all 4400 participants)	41.4 (38.2 to 44.6)
Attended a village health nutrition and sanitation day in the past month (*n* = all 4400 participants)	45.7 (42.3 to 49.3)
Growth monitoring for child received in the past year (*n* = 3300 mothers of children aged 0–23 months)	73.7 (70.2 to 76.9)
Application used by *Anganwadi* workers in the district^c^ (*n* = all 4400 participants)	60.6 (57.0 to 64.1)
**Messages received about pregnancy nutrition (*n* = 1100 pregnant women)**	
Take one iron-folic acid tablet at night after dinner	84.4 (80.6 to 87.5)
Take one iron-folic acid tablet daily for at least 100 days	65.1 (61.4 to 68.6)
Consume green and yellow/orange coloured fruits and vegetables and drink milk daily	93.7 (91.3 to 95.4)
Increase the quantity of food during pregnancy	79.2 (76.0 to 82.0)
Take frequent meals during the day (five to six small meals rather than three)	86.7 (83.4 to 89.4)
Rest for 1 to 2 hours in a day	86.8 (83.9 to 89.3)
Non-vegetarians should include non-vegetarian items in the diet	62.1 (58.1 to 66.0)
**Infant and young child nutrition **	
Feed colostrum immediately after birth (*n* = 1100 pregnant women)	62.9 (59.0 to 66.7)
Breastfeed the baby within 1 hour of birth (*n* = all 4400 participants)	79.6 (76.5 to 82.4)
Exclusive breastfeeding until 6 months of age (*n* = all 4400 participants)	83.8 (81.1 to 86.1)
Initiate complementary feeding at age 6 months, along with breastfeeding (*n* = 3300 mothers of children aged 0–23 months)	86.5 (83.2 to 89.2)
From 6–8 months of age, child should be fed two bowls of complementary foods daily (*n* = 3300 mothers of children aged 0–23 months)	76.4 (73.0 to 79.6)
From 9–11 months of age, child should be fed three bowls of complementary foods daily (*n* = 3300 mothers of children aged 0–23 months)	66.6 (63.0 to 70.1)
From 12–24 months of age, child should be fed four bowls of complementary foods daily (*n* = 3300 mothers of children aged 0–23 months)	63.2 (59.1 to 67.0)
Feed the child from a separate bowl (*n* = 3300 mothers of children aged 0–23 months)	67.1 (63.3 to 70.6)
Play with the child while feeding (*n* = 3300 mothers of children aged 0–23 months)	74.7 (70.4 to 78.5)
After a child's illness, increase the quantity of food fed (*n* = 3300 mothers of children aged 0–23 months)	66.8 (63.1 to 70.4)
Wash hands before preparing food and feeding the baby (*n* = 3300 mothers of children aged 0–23 months)	87.9 (84.3 to 90.8)
**Practices**	
Consumed iron-folic acid tablets for 100 or more days during pregnancy (*n* = 3300 mothers of children aged 0–23 months)	57.6 (54.3 to 60.9)
Achieved the minimum dietary diversity for pregnant women (≥ five food groups; (*n* = 1100 pregnant women)	67.4 (63.0 to 71.6)
Breastfeeding initiated in the first hour of life (*n* = 3300 mothers of children aged 0–23 months)	67.4 (64.5 to 70.2)
Child exclusively breastfed in the first 6 months of life (*n* = 1100 children)	81.0 (76.6 to 84.7)
Receiving solid or semi-solid food and breast milk (*n* = 517 children aged 6–8 months)	61.2 (55.5 to 66.6)
Minimum dietary diversity (*n* = 2200 children aged 6–23 months)	35.6 (31.8 to 39.6)
Minimum meal frequency (*n* = 2200 children aged 6–23 months)	60.7 (56.1 to 65.1)
Minimum acceptable diet (*n* = 2200 children aged 6–23 months)	26.3 (23.0 to 30.0)
Increase food quantity or breastfeeding following illness (*n* = 3300 mothers of children aged 0–23 months)	41.5 (37.8 to 45.3)

CI: confidence interval.

^a^ Proportion is survey weighted and confidence intervals are adjusted for clustered sampling design.

^b^ Missing data for the number of days of take-home rations for nine pregnant women, 39 women with children ages 0–5 months, and 51 women with children ages 6–23 months.

^c^ Refers to common application software of the integrated child development activities.

Core scheme messages (i.e. consumption of iron-folic acid after dinner during pregnancy; consumption of green and yellow/orange fruits and vegetables during pregnancy; breastfeeding in the first hour of life; exclusive breastfeeding for the first 6 months of life; and age-appropriate complementary feeding at age 6 months) had been heard by 80% or more of respondents (Table 2). More specific messages about nutrition reached somewhat fewer beneficiaries. The least heard messages were that non-vegetarian pregnant women should include non-vegetarian items in their diet (62.1%; 95% CI: 58.1 to 66.0); colostrum should be fed immediately after birth (62.9%; 95% CI: 59.0 to 66.7); and children aged 12–24 months should receive four bowls of complementary foods daily (63.2%; 95% CI: 59.1 to 67.0; Table 2).

The most common nutrition practice was exclusive breastfeeding in the first 6 months of life (81.0%; 95% CI: 76.6 to 84.7). The least common practice was the achievement of a minimum acceptable diet for children aged 6 to 23 months (26.3%; 95% CI: 23.0 to 30.0). Achievement of the minimum diet diversity was considerably higher among pregnant women (67.4%; 95% CI: 63.0 to 71.6) than among children aged 6 to 23 months (35.6%; 95% CI: 31.8 to 39.6). Approximately two out of five women did not consume more than 100 days’ worth of iron-folic acid tablets during pregnancy, and a similar proportion did not initiate complementary feeding when their baby reached 6 months of age. Relative to most of the practices, increasing the quantity of food or breastfeeding following an illness was practiced by fewer respondents (41.5%; 95% CI: 37.8 to 45.3; Table 2).

After controlling for potential sociodemographic confounders, the receipt of scheme activities was frequently associated with having heard key nutrition messages. Among 110 regression models testing unique pairs of activities and messages, 103 showed a statistically significant positive relationship (Table 3). Among the seven activities, relatively strong associations were observed (with nutrition messages received as the outcome) for growth monitoring (median RR: 1.30); nutrition information received during pregnancy (median RR: 1.25); and having ever attended a community-based event (median RR: 1.19; available in the online repository).^13^ More specific nutrition messages (which were heard by a relatively smaller proportion of respondents) typically showed larger improvements associated with the receipt of activities. For example, across all the relevant activities that were tested, the median RR was above 1.20 for the following messages: take one iron-folic acid tablet daily for at least 100 days during pregnancy; non-vegetarians should include non-vegetarian items in the diet; from 9–11 months of age, the child should be fed three bowls of complementary food daily; from 12–24 months of age, the child should be fed four bowls of complementary food daily; after a child’s illness, increase the quantity of food fed (online repository).^13^

**Table 3:** The association between activities of the Integrated Child Development Services scheme and messages received, India, 2021

Nutrition message	Service received, RR (95% CI)						
	Take-home rations for more than 21 days^a,b^	Nutrition information from an *Anganwadi* worker during pregnancy	Home visit by an *Anganwadi* worker in past month (*n* = 1100 pregnant women)	Ever attended a community-based event^c^	Attended the village health sanitation and nutrition day in the past month	Growth monitoring in past year (*n* = 3300 women with children aged 0–23 months)	Application^d^ used by *Anganwadi* worker
**Pregnancy nutrition (*n* = 1100 pregnant women)**							
Take one iron-folic acid tablet at night after dinner	1.05 (1.01 to 1.10)	1.25 (1.18 to 1.32)	1.13 (1.07 to 1.20)	1.13 (1.09 to 1.18)	1.06 (1.01 to 1.11)	NA	1.02 (0.97 to 1.07)
Take one iron-folic acid tablet daily for at least 100 days	1.23 (1.14 to 1.33)	1.39 (1.26 to 1.52)	1.22 (1.11 to 1.33)	1.35 (1.26 to 1.45)	1.14 (1.06 to 1.24)	NA	1.13 (1.04 to 1.24)
Consume green and yellow/orange coloured fruits and vegetables and drink milk daily	1.05 (1.02 to 1.08)	1.10 (1.06 to 1.13)	1.03 (0.99 to 1.06)	1.05 (1.03 to 1.08)	1.01 (0.98 to 1.04)	NA	1.03 (1.00 to 1.07)
Increase the quantity of food during pregnancy	1.13 (1.07 to 1.19)	1.24 (1.16 to 1.33)	1.11 (1.05 to 1.19)	1.10 (1.04 to 1.15)	1.04 (0.98 to 1.09)	NA	1.08 (1.01 to 1.15)
Take frequent meals during the day (five to six small meals rather than three)	1.08 (1.03 to 1.13)	1.16 (1.10 to 1.23)	1.10 (1.04 to 1.16)	1.10 (1.06 to 1.15)	1.03 (0.99 to 1.08)	NA	1.01 (0.96 to 1.06)
Rest for 1–2 hours in a day	1.07 (1.03 to 1.12)	1.16 (1.11 to 1.22)	1.08 (1.03 to 1.13)	1.14 (1.10 to 1.17)	1.08 (1.04 to 1.12)	NA	1.05 (1.00 to 1.10)
Non-vegetarians should include non-vegetarian items in the diet	1.21 (1.11 to 1.32)	1.43 (1.29 to 1.59)	1.28 (1.16 to 1.42)	1.30 (1.20 to 1.41)	1.10 (1.01 to 1.20)	NA	1.08 (0.98 to 1.19)
**Infant and young child nutrition**							
Feed colostrum immediately after birth (*n* = 1100 pregnant women)	1.15 (1.05 to 1.25)	1.44 (1.29 to 1.60)	1.21 (1.10 to 1.33)	1.36 (1.26 to 1.47)	1.20 (1.10 to 1.31)	NA	1.12 (1.02 to 1.23)
Breastfeed the baby within 1 hour of birth (*n* = 4400 survey participants)	1.08 (1.05 to 1.11)	1.25 (1.21 to 1.30)	1.19 (1.10 to 1.28)	1.17 (1.15 to 1.20)	1.10 (1.08 to 1.13)	1.20 (1.15 to 1.26)	1.14 (1.11 to 1.18)
Exclusive breastfeeding until 6 months of age (*n* = 4400 survey participants)	1.05 (1.03 to 1.08)	1.17 (1.13 to 1.20)	1.13 (1.05 to 1.21)	1.10 (1.08 to 1.13)	1.06 (1.03 to 1.08)	1.15 (1.11 to 1.19)	1.06 (1.03 to 1.09)
Initiate complementary feeding at age 6 months, along with breastfeeding (*n* = 3300 women with children aged 0–23 months)	1.05 (1.03 to 1.08)	1.15 (1.12 to 1.18)	NA	1.10 (1.08 to 1.13)	1.07 (1.05 to 1.09)	1.17 (1.13 to 1.22)	1.07 (1.05 to 1.10)
From 6–8 months of age, child should be fed two bowls of complementary foods daily (*n* = 3300 women with children aged 0–23 months)	1.09 (1.05 to 1.12)	1.22 (1.17 to 1.27)	NA	1.21 (1.18 to 1.25)	1.12 (1.08 to 1.15)	1.28 (1.21 to 1.35)	1.12 (1.08 to 1.17)
From 9–11 months of age, child should be fed three bowls of complementary foods daily (*n* = 3300 women with children aged 0–23 months)	1.15 (1.10 to 1.20)	1.33 (1.26 to 1.40)	NA	1.34 (1.29 to 1.40)	1.22 (1.17 to 1.27)	1.35 (1.26 to 1.44)	1.13 (1.07 to 1.18)
From 12–24 months of age, child should be fed four bowls of complementary foods daily (*n* = 3300 women with children aged 0–23 months)	1.19 (1.14 to 1.25)	1.29 (1.22 to 1.36)	NA	1.37 (1.31 to 1.43)	1.25 (1.20 to 1.31)	1.37 (1.28 to 1.48)	1.13 (1.07 to 1.19)
Feed the child from a separate bowl (*n* = 3300 women with children aged 0–23 months)	1.12 (1.07 to 1.16)	1.35 (1.28 to 1.42)	NA	1.33 (1.27 to 1.38)	1.17 (1.12 to 1.22)	1.39 (1.30 to 1.49)	1.16 (1.10 to 1.21)
Play with the child while feeding (*n* = 3300 women with children aged 0–23 months)	1.09 (1.06 to 1.13)	1.23 (1.18 to 1.28)	NA	1.22 (1.18 to 1.26)	1.11 (1.08 to 1.15)	1.31 (1.24 to 1.39)	1.11 (1.06 to 1.15)
After a child's illness, increase the quantity of food fed (*n* = 3300 women with children aged 0–23 months)	1.09 (1.04 to 1.14)	1.32 (1.25 to 1.40)	NA	1.28 (1.23 to 1.34)	1.20 (1.15 to 1.25)	1.40 (1.31 to 1.51)	1.15 (1.09 to 1.21)
Wash hands before preparing food and feeding the baby (*n* = 3300 women with children aged 0–23 months)	1.05 (1.03 to 1.07)	1.12 (1.09 to 1.16)	NA	1.10 (1.08 to 1.12)	1.05 (1.03 to 1.07)	1.15 (1.11 to 1.20)	1.03 (1.01 to 1.06)

CI: confidence interval; NA: not applicable; RR: risk ratio.

^a^ Take-home rations comprise of a variety of products, dry rations and hot cooked meals, depending on the implementation locality.

^b^ Missing data for the number of days of take-home rations for nine pregnant women, 39 women with children aged 0–5 months, and 51 women with children aged 6–23 months.

^c^ A community-based event is a group celebration of milestones in the mother or child’s life which includes nutrition messages.

^d^ Application refers to a common application software of the integrated child development activities.

Note: We set integrated child development activities as independent variable and messages received as dependent variable, and adjusted for sociodemographic factors.

Regarding the association between activities provided and nutrition practices, a statistically significant positive association was observed among 39 out of 54 tested service–practice pairs (Table 4). Information received from the *Anganwadi* worker during pregnancy demonstrated the strongest association with practices (median RR: 1.32; available in the online repository).^13^ Having attended a community-based event and having received growth monitoring in the past year were also associated with substantial impacts (median RR: 1.21 for both activities).^13^ Across all seven activities, the nutrition practices that were associated with the largest improvements were minimum acceptable diet (median RR: 1.69); and minimum dietary diversity (median RR: 1.63) among children. Appropriate pregnancy diet practices (iron-folic acid consumption for more than 100 days and achievement of the minimum dietary diversity) were also significantly higher among service recipients (median RR: 1.23).

**Table 4:** The association between activities of the Integrated Child Development Services scheme and nutrition practices, India, 2021

Nutrition practice	Intervention received, RR (95% CI)						
	Take-home rations for more than 21 days^a,b^	Nutrition information from *Anganwadi* worker during pregnancy	Home visit by an *Anganwadi* worker in past month (*n* = 1100 pregnant women)	Ever attended a community-based event^c^	Attended the village health sanitation and nutrition day in the past month	Growth monitoring in past year (*n* = 3300 women with children aged 0–23 months)	Application^d^ used by *Anganwadi* worker
Consumed iron-folic acid tablets for 100 or more days during pregnancy (*n* = 3300 women with children aged 0–23 months)	1.14 (1.08 to 1.20)	1.37 (1.29 to 1.47)	NA	1.22 (1.16 to 1.28)	1.22 (1.16 to 1.29)	1.43 (1.31 to 1.55)	1.23 (1.16 to 1.31)
Achieved the minimum dietary diversity for pregnant women (≥ five food groups; *n* = 1100 pregnant women)	1.20 (1.10 to 1.31)	1.57 (1.41 to 1.74)	1.35 (1.22 to 1.49)	1.24 (1.15 to 1.34)	1.21 (1.12 to 1.31)	NA	1.00 (0.92 to 1.09)
Breastfeeding initiated in the first hour of life (*n* = 3300 women with children aged 0–23 months)	1.08 (1.03 to 1.13)	1.15 (1.09 to 1.21)	NA	1.17 (1.12 to 1.22)	1.09 (1.04 to 1.14)	1.15 (1.08 to 1.22)	1.02 (0.97 to 1.07)
Child exclusively breastfed in the first 6 months of life (*n* = 1100 children aged 0–5 months)	1.07 (1.01 to 1.13)	1.07 (1.01 to 1.14)	NA	1.07 (1.02 to 1.13)	1.03 (0.97 to 1.08)	1.03 (0.98 to 1.09)	1.06 (1.00 to 1.12)
Children aged 6–8 months of age receiving solid or semi-solid food and breast milk (*n* = 517)	0.96 (0.84 to 1.10)	1.32 (1.14 to 1.53)	NA	1.21 (1.07 to 1.37)	1.01 (0.89 to 1.15)	1.14 (0.94 to 1.38)	0.98 (0.85 to 1.12)
Minimum dietary diversity (*n* = 2200 children aged 6–23 months)	1.42 (1.26 to 1.59)	1.78 (1.55 to 2.06)	NA	1.75 (1.57 to 1.96)	1.51 (1.36 to 1.69)	2.20 (1.72 to 2.82)	1.21 (1.07 to 1.37)
Minimum meal frequency (*n* = 2200 children aged 6–23 months)	1.02 (0.96 to 1.10)	1.19 (1.10 to 1.28)	NA	1.16 (1.09 to 1.24)	1.08 (1.01 to 1.15)	1.20 (1.07 to 1.33)	0.95 (0.89 to 1.02)
Minimum acceptable diet (*n* = 2200 children aged 6–23 months)	1.36 (1.18 to 1.57)	1.80 (1.51 to 2.14)	NA	1.83 (1.59 to 2.11)	1.57 (1.37 to 1.80)	2.30 (1.70 to 3.11)	1.12 (0.97 to 1.30)
Increase food quantity or breastfeeding following illness (*n* = 3300 women with children aged 0–23 months)	1.09 (1.00 to 1.18)	1.20 (1.10 to 1.32)	NA	1.19 (1.09 to 1.29)	1.07 (0.99 to 1.17)	1.22 (1.09 to 1.37)	1.02 (0.93 to 1.11)

CI: confidence interval; NA: not applicable; RR: risk ratio.

^a^ Take-home rations comprise of a variety of products, dry rations and hot cooked meals, depending on the implementation locality.

^b^ Missing data for the number of days of take-home rations for nine pregnant women, 39 women with children aged 0–5 months, and 51 women with children aged 6–23 months.

^c^ A community-based event is a group celebration of milestones in the mother or child’s life which includes nutrition messages.

^d^ Application refers to a common application software of the integrated child development activities.

Note: We set integrated child development activities as independent variable and messages received as dependent variable, and adjusted for sociodemographic factors.

The association between community-based events and all seven nutrition practices was significantly mediated via nutrition messages (median proportion mediated: 18.7%; *P*-values < 0.01 for all seven practices; Table 5 and online repository).^13^ For nutrition information from *Anganwadi* workers during pregnancy, significant mediation by nutrition messages was seen for six out of seven nutrition practices (median proportion mediated: 20.9%; *P*-values < 0.01 for six out of seven practices; Table 5 and online repository).^13^

**Table 5:** Percentage of the association between activities of Integrated Child Development Services scheme and nutrition practice that is explained by having received related nutritional messages, India, 2021

Nutrition practice	Service received, % (95% CI)						
	Take-home rations for more than 21 days^a,b^	Nutrition information from *Anganwadi* worker during pregnancy	Home visit by *Anganwadi* worker in past month (*n* = 1100 pregnant women)	Ever attended a community-based event^c^	Attended the village health sanitation and nutrition day in the past month	Growth monitoring in past year (*n* = 3300 women with children aged 0–23 months)	Application^d^ used by *Anganwadi* worker
Achieved the minimum dietary diversity for pregnant women (≥ five food groups; *n* = 1100 pregnant women)	19.6 (13.5 to 38.5)	14.9 (11.7 to 20.4)	3.6 (2.5 to 6.0)	17.6 (13.1 to 27.5)	−2.4 (−5.3 to −1.6)	NA	13.9 (−100.0 to 100.0)
Breastfeeding initiated in the first hour of life (*n* = 3300 women with children aged 0–23 months)	17.3 (11.2 to 39.0)	44.2 (31.2 to 75.6)	NA	35.1 (23.7 to 72.2)	24.2 (16.2 to 50.5)	37.2 (23.5 to 87.6)	56.0 (24.9 to 100.0)
Child exclusively breastfed in the first 6 months of life (*n* = 1100 children aged 0–5 months)	13.0 (6.4 to 60.2)	20.9 (−89.2 to 100.0)	NA	8.9 (5.5 to 24.8)	5.2 (−50.8 to 73.8)	17.4 (−100.0 to 100.0)	−0.2 (−0.7to −0.1)
Children aged 6–8 months of age receiving solid or semi-solid food and breast milk (*n* = 517)	3.1 (−33.1 to 36.6)	22.4 (15.4 to 41.4)	NA	27.4 (18.1 to 56.3)	71.6 (−100.0 to 100.0)	32.4 (−100.0 to 100.0)	−42.7 (−100.0 to 100.0)
Minimum dietary diversity (*n* = 2200 children aged 6–23 months)	6.2 (4.6 to 9.4)	12.0 (9.5 to 17.0)	NA	8.8 (7.4 to 11.4)	11.3 (8.9 to 16.1)	10.9 (8.7 to 15.4)	17.4 (11.4 to 40.1)
Minimum meal frequency (*n* = 2200 children aged 6–23 months)	11.4 (−100.0 to 100.0)	14.9 (9.9 to 32.0)	NA	11.5 (8.2 to 20.3)	31.1 (−100.0 to 100.0)	17.0 (10.4 to 48.3)	−46.1 (−100.0 to 100.0)
Increase food quantity or breastfeeding following illness (*n* = 3300 women with children aged 0–23 months)	44.8 (−100.0 to 100.0)	34.6 (21.8 to 93.8)	NA	37.7 (24.2 to 88.7)	41.3 (21.4 to 100.0)	36.9 (22.4 to 100.0)	38.0 (−100.0 to 100.0)

CI: confidence interval; NA: not applicable.

^a^ Take-home rations comprise of a variety of products, dry rations and hot cooked meals, depending on the implementation locality.

^b^ Missing data for the number of days of take-home rations for nine pregnant women, 39 women with children aged 0–5 months, and 51 women with children aged 6–23 months.

^c^ A community-based event is a group celebration of milestones in the mother or child’s life which includes nutrition messages.

Note: We set integrated child development activities as independent variable and messages received as dependent variable, and adjusted for sociodemographic factors.

## Discussion

This study of pregnant women and mothers of young children enrolled in the Integrated Child Development Services scheme suggests that one of the world’s largest nutrition programmes may be effectively improving beneficiary knowledge and practices. A positive association was consistently observed between seven scheme activities and a range of nutrition knowledge and nutrition practice indicators. Furthermore, a statistically significant proportion of the association between several activities and nutrition practices was mediated by knowledge. Three activities showed a greater advantage for the beneficiaries: receipt of nutrition information from *Anganwadi* workers during pregnancy; participation in community-based events; and receipt of growth monitoring activities.

In 2015–2016, 71.8% of scheme beneficiaries reported having received health and nutrition counselling in pregnancy,^15^ which is higher than our estimate of 62.3%. This result could be explained by the fact that we conducted the study when the COVID-19 pandemic greatly burdened India. Group events, such as community-based events and village health sanitation and nutrition days, were reduced and modified during the pandemic, which could explain why the respondents used these activities the least. While this result suggests that COVID-19 may have somewhat constrained the delivery of activities, the overall service delivery levels across all indicators remained relatively strong given the challenging operational conditions.

Nutrition activities that include a strong component of nutritional messaging were associated with the largest impacts on both nutrition knowledge and practices. *Anganwadi* workers are trained to provide a holistic curriculum of nutrition messages to beneficiaries during pregnancy and during the first two years of their children’s life. Other activities that do not include a strong interactive counselling component, such as delivery of take-home rations and attendance at village health sanitation and nutrition days, were less associated with improved knowledge or practices. The modest associations between use of the mobile-based application and nutrition knowledge and practices may have been due to ongoing problems with the application’s functionality.

All of the nutrition messages examined in this study had been heard by more than three out of five beneficiaries, which represents an important reach of the scheme. Messages which had been heard by a lower proportion of beneficiaries tended to have larger improvements associated with activities. This result reinforces the importance of scheme service delivery for enhancing these lagging messages.

Having heard relevant nutrition messages was a statistically significant mediator of improved nutrition practices. The linkage between nutrition knowledge and practice is an underlying assumption of nutrition education programmes. However, knowledge is necessary but not sufficient to drive behaviour change.^23^ In this study, the proportion of the total effect mediated was relatively low, which could be explained by several potential factors, such as how knowledge was measured and included in the statistical models (further explanation in online repository).^13^

A large meta-analysis found that social mobilization and behaviour change communication were linked to a 73% increase in the odds of exclusive breastfeeding, but no statistically significant impacts on early initiation of breastfeeding, minimum dietary diversity or minimum meal frequency.^24^ By contrast, the 63% median increase in child dietary diversity and 69% median increase in minimum acceptable diet associated with scheme activities in this study are considerable. Although the association of scheme activities with exclusive breastfeeding in this study are smaller than that reported in the meta-analysis, the high overall prevalence of exclusive breastfeeding in the present study provides a mathematical constraint to the magnitude of the RR. Another review, which primarily included randomized controlled trials, found that breastfeeding counselling was associated with a 20% increase in the early initiation of breastfeeding.^25^ The increases in early initiation of breastfeeding associated with counselling-intensive activities in the present study are similar in magnitude to those reported in the review. The evidence from this study therefore suggests that effects in large-scale programmes could potentially attain the effects seen in the typically smaller, more controlled settings of interventional research.

This study has limitations. For the safety of study participants and research personnel in the context of high COVID-19 transmission, data for this study were collected by telephone. Hence, collecting anthropometric or hematologic indicators of nutritional status was impossible. However, a large body of evidence supports the fact that improvements in infant and young child feeding practices are linked to better health outcomes.^26^ We therefore believe it is plausible to expect that the improvements in nutrition practices observed in this study would result in some improvement in child outcomes.

The potential for confounding and measurement error should be considered when interpreting the results of this study. As for all observational research, there exists a risk of unmeasured confounding. To reduce bias in the estimates of the association between nutrition activities and knowledge or practices, we statistically controlled for seven socioeconomic variables. However, one or multiple unmeasured confounders might exist, which could bias our reported associations. Participants self-reported service receipt, nutrition knowledge and practices, which could result in misclassification due to inaccurate recall. If misclassification of the exposure and outcome are independent in a given model, then the RR would be biased towards the null. The reader should therefore be duly prudent when considering the causal implications of these findings. Other potential limitations, such as the use of a cross-sectional design and telephonic data collection, are presented in the online repository.^13^

In India, improved nutrition practices can likely be achieved through expansion and intensification of access to counselling-intensive interventions through the Integrated Child Development Services scheme. Since knowledge is a mediator of improved practices, the scheme may consider focusing on increasing the dissemination of messages and ensuring that beneficiary recall and comprehension of the messages is firm. Policy-makers worldwide may use the data presented here to adapt aspects of the scheme’s activities that are appropriate for other contexts, as the evidence suggests the scheme has yielded benefits for nutrition knowledge and practices among beneficiaries.
